# Discovery of a novel filamentous prophage in the genome of the *Mimosa pudica* microsymbiont *Cupriavidus taiwanensis* STM 6018

**DOI:** 10.3389/fmicb.2023.1082107

**Published:** 2023-02-28

**Authors:** Agnieszka Klonowska, Julie Ardley, Lionel Moulin, Jaco Zandberg, Delphine Patrel, Margaret Gollagher, Dora Marinova, T. B. K. Reddy, Neha Varghese, Marcel Huntemann, Tanja Woyke, Rekha Seshadri, Natalia Ivanova, Nikos Kyrpides, Wayne Reeve

**Affiliations:** ^1^Université de Montpellier, IRD, CIRAD, INRAE, Institut AgroPHIM Plant Health Institute, Montpellier, France; ^2^Centre for Crop and Food Innovation, Food Futures Institute, College of Science, Health, Engineering and Education, Murdoch University, Murdoch, WA, Australia; ^3^Curtin University Sustainability Policy Institute, Curtin University, Bentley, WA, Australia; ^4^Department of Energy Joint Genome Institute, Lawrence Berkeley National Laboratory, Berkeley, CA, United States

**Keywords:** *Betaproteobacteria*, root-nodule bacteria, bacteriophage, filamentous phage, symbiosis

## Abstract

Integrated virus genomes (prophages) are commonly found in sequenced bacterial genomes but have rarely been described in detail for rhizobial genomes. *Cupriavidus taiwanensis* STM 6018 is a rhizobial *Betaproteobacteria* strain that was isolated in 2006 from a root nodule of a *Mimosa pudica* host in French Guiana, South America. Here we describe features of the genome of STM 6018, focusing on the characterization of two different types of prophages that have been identified in its genome. The draft genome of STM 6018 is 6,553,639 bp, and consists of 80 scaffolds, containing 5,864 protein-coding genes and 61 RNA genes. STM 6018 contains all the nodulation and nitrogen fixation gene clusters common to symbiotic *Cupriavidus* species; sharing >99.97% bp identity homology to the *nod*/*nif*/*noeM* gene clusters from *C. taiwanensis* LMG19424^T^ and “*Cupriavidus neocalidonicus”* STM 6070. The STM 6018 genome contains the genomes of two prophages: one complete Mu-like capsular phage and one filamentous phage, which integrates into a putative *dif* site. This is the first characterization of a filamentous phage found within the genome of a rhizobial strain. Further examination of sequenced rhizobial genomes identified filamentous prophage sequences in several Beta-rhizobial strains but not in any Alphaproteobacterial rhizobia.

## Introduction

Root nodule bacteria, collectively known as rhizobia, are the dinitrogen-fixing microsymbionts of legumes. Their taxonomic diversity encompasses both the *Alpha-* and *Beta-* subclasses of *Proteobacteria*, and the names Alpha- and Beta-rhizobia are used for convenience to distinguish each group (Moulin et al., 2001; Gyaneshwar et al., 2011). To date, Beta-rhizobia include three genera: *Paraburkholderia*, *Cupriavidus* (formerly *Ralstonia*; Vandamme and Coenye, 2004) and *Trinickia* (Estrada-de los Santos et al., 2018; de Lajudie et al., 2019). Beta-rhizobia have a particular affinity for the genus *Mimosa* and related South American mimosoid legumes and nodulate at least 68 *Mimosa* species, including in particular the invasive species *M. pudica*, *M. pigra*, and *M. bimucronata* in Asia, Australia, and Central and South America (Barrett and Parker, 2005; Parker et al., 2007; Bontemps et al., 2010; dos Reis Jr et al., 2010; Gehlot et al., 2013; Estrada-de los Santos et al., 2018). Based on a comparison of house-keeping and nodulation gene phylogenies, *Paraburkholderia* species have been postulated to be ancestral symbionts of South American *Mimosa* and Piptadenia group species (Bontemps et al., 2010; Bournaud et al., 2013), while symbiotic *Cupriavidus* appear to have recently acquired nodulation genes from a *Paraburkholderia* ancestor (Chen et al., 2003; da Silva et al., 2012; Mishra et al., 2012). *Cupriavidus* symbionts have been isolated from invasive *M. pudica* and *M. diplotricha* in Central America, China, Christmas Island, French Guiana, India, New Caledonia and Taiwan (Chen et al., 2003; Andam et al., 2007; Klonowska et al., 2012; Mishra et al., 2012; Gehlot et al., 2013; De Meyer et al., 2018; Liu et al., 2020), as well as from native *Mimosa* spp. in southern United States and Uruguay (Andam et al., 2007; Platero et al., 2016) and *Parapiptadenia rigida* in Uruguay (Taulé et al., 2012).

Whether *Paraburkholderia* or *Cupriavidus* strains are the dominant nodule occupants of these mimosoid hosts appears to depend primarily on edaphic factors, and to some extent on host preference. In general, hosts growing in acidic soils are nodulated by *Paraburkholderia* strains, whereas *Cupriavidus* predominates in soils that are neutral-alkaline and/or have high heavy metal content (Klonowska et al., 2012; Mishra et al., 2012; Platero et al., 2016; Liu et al., 2020). Competition studies have revealed the dominance of *Paraburkholderia phymatum* and *Paraburkholderia tuberum* within *M. pudica* nodules compared to *Cupriavidus* or *Rhizobium* strains, however, the Taiwanese accession *M. pudica* var. *unijuga* displayed a higher affinity for *C. taiwanensis* strains, suggesting local co-adaptation (Melkonian et al., 2014).

Recent analyses of non-symbiotic *Paraburkholderia* and *Cupriavidus* strains have identified diverse integrated bacteriophages (prophages) that comprise a considerable portion of some of their genomes (Pratama et al., 2018; Van Houdt et al., 2018). Bacteriophages are ubiquitous in bacterial populations, and although they can kill or impose metabolic burdens on their hosts, phages that are integrated into the chromosome can supply benefits to the host bacterium as agents of horizontal gene transfer (HGT), sources of genetic variation, in bacterial competition, and by supplying phage-encoded virulence genes; they may also affect bacterial motility, biofilm production and relationships with hosts (Figueroa-Bossi et al., 2001; Wagner and Waldor, 2002; Allison, 2007; Harrison and Brockhurst, 2017; Secor et al., 2020). Although the recent expansion of both phage-derived and bacterial sequence databases has uncovered a high abundance of prophages within Proteobacterial genomes in particular (López-Leal et al., 2022), their role in rhizobial lifestyles is currently not well understood (Ford et al., 2021).

*Cupriavidus taiwanensis* strain STM 6018 was isolated from a *M. pudica* trap host using a soil sample from near the town of Remire, French Guiana, South America (Mishra et al., 2012) and was selected for sequencing at the US Department of Energy Joint Genome Institute (JGI) as part of the Genomic Encyclopedia of Bacteria and Archaea-Root Nodule Bacteria (GEBA-RNB) project (Reeve et al., 2015). Preliminary analysis of the genome identified that it had the unusual property of containing two prophages, one being a capsular phage and the other a filamentous phage. Here we describe the symbiotic and genomic features of STM 6018, and specifically focus on characterizing the two prophages and examining features in the STM 6018 genome that are putatively important for phage interactions with this host. We furthermore survey the genomes of other rhizobial strains for the presence of filamentous prophages and compare the STM 6018 filamentous prophage with those found in two other Beta-rhizobia strains.

## Results and discussion

### STM 6018 morphology and growth, genome classification, and species assignment

*C. taiwanensis* STM 6018 is a motile, Gram-negative rod (Supplementary Figures S1A,B) in the order *Burkholderiales* of the class *Betaproteobacteria*. It is fast growing, forming colonies within 3–4 days when grown on half strength Lupin Agar (½LA; Howieson et al., 1988), tryptone-yeast extract agar (TY; Beringer, 1974) or a modified yeast-mannitol agar (YMAm; Howieson and Dilworth, 2016) at 28°C. Colonies on ½LA are white-opaque, slightly domed and moderately mucoid with smooth margins (Supplementary Figure S1C).

The STM 6018 genome project is deposited in the Genomes OnLine Database (GOLD; Mukherjee et al., 2020) and a draft genome sequence is available in the Integrated Microbial Genomes System (Chen et al., 2021). Minimum Information about the Genome Sequence (MIGS) is provided in Supplementary Table S1. Phylogenetic analysis based on an intragenic fragment of the 16S rRNA gene shows that the *C. taiwanensis* strains STM 6018 and LMG 19424^T^ are most closely related to *Cupriavidus nantongensis* X1^T^*, Cupriavidus alkaliphilus* ASC-732^T^ and the rhizobial strain “*Cupriavidus neocaledonicus*” STM 6070 (Supplementary Figure S2). The STM 6018 16S rRNA gene shares 99.6, 99.37, 99.14, 99.02 and 98.5% sequence identity (over 1,426 bp) to the 16S rRNA genes of the type strains *C. taiwanensis* LMG19424^T^ (Amadou et al., 2008), *C. nantongensis* X1^T^ (Sun et al., 2016), “*C. neocaledonicus*” STM 6070 (Klonowska et al., 2020), *C. alkaliphilus* ASC-732^T^ (Estrada-de los Santos et al., 2012) and *C. necator* ATCC43291^T^ (N-1; Poehlein et al., 2011), respectively.

The species assignment for STM 6018 was further assessed by calculating the average nucleotide identity (ANI) values of this genome to other *Cupriavidus* genomes (Table 1). Analysis of the ANIb and ANIm values (>98%, over 92% conserved DNA) and ANIg values (>99%) showed that STM 6018 belongs to the same species as the *M. pudica*-nodulating *C. taiwanensis* LMG19424^T^, consistent with the 16S rRNA gene analysis. The next closest rhizobial species is “*Cupriavidus neocaledonicus”* STM 6070, also a microsymbiont of *M. pudica* (Klonowska et al., 2020), with ANIb and ANIm values (>93% over >82% conserved DNA) and ANIg values (>94%). In contrast, the sequenced rhizobial strains *Cupriavidus* sp. AMP6, isolated from native *Mimosa asperata* in Texas (Andam et al., 2007) and *C. necator* UYPR2.512, which nodulates *Parapiptadenia rigida* in Uruguay (Taulé et al., 2012), have ANIb, ANIm or ANIg values <90%.

**Table 1:** Average nucleotide identities^*^ (ANI) of STM6018 genome compared to close relatives.

	STM6018				
Genomes	ANIm^*^	% Identical DNA	ANIb^*^	% Identical DNA	ANIg^*^
*Cupriavidus taiwanensis* LMG19424^T^ (PRJNA61615)	98.90	95.16	98.82	92.6	99.03
*“Cupriavidus neocaledonicus”* STM6070 (PRJNA199025)	93.99	85.39	93.40	82.59	94.68
*Cupriavidus necator* H16 (PRJNA158697)	89.60	62.24	87.39	63.84	89.73
*Cupriavidus necator* N-1^T^ (PRJNA68689)	89.55	53.43	86.62	56.09	89.61
*Cupriavidus necator* UYPR2.512 (PRJNA199176)	89.53	56.61	87.23	57.9	89.68
*Cupriavidus* sp. AMP6 (PRJNA195776)	88.93	53.80	85.71	56.52	88.44
*Cupriavidus metallidurans* CH34^T^ (PRJNA57815)	85.77	25.58	78.67	43.17	81.39
*Ralstonia solanacearum* GMI1000 (PRJNA57593)	85.08	18.80	76.57	30.41	79.49
*Ralstonia pickettii* 12D (PRJNA58859)	84.55	14.30	74.95	31.23	77.58

* Average Nucleotide Identities (ANI) were computed from whole genome alignments of *Cupriavidus* and *Ralstonia*. The ANI values were computed using MUMmer (ANIm) or with the BLASTN (ANIb) algorithm as implemented in jSpecies (Goris et al., 2007) or as pairwise bidirectional best nSimScan hits (ANIg) (Varghese et al., 2015).

T Indicates type strains of the species.

### General properties and features of the STM 6018 genome

The STM 6018 draft genome is given as 6,553,639 nucleotides with 66.90% GC content (Supplementary Table S2) and comprised of 80 scaffolds of 80 contigs (449x sequence coverage) with a total of 5,925 annotated genes, of which 5,864 are protein encoding and 61 RNA only encoding genes. Most of the protein encoding genes were predicted to have functions (80.69%), while the remaining genes were annotated as hypothetical. The distribution of genes into functional COG categories is presented in Supplementary Table S3.

### Pangenome analysis: Comparisons of STM 6018 with other sequenced symbiotic Cupriavidus genomes

A pangenome analysis comparing the STM 6018 genome to the genomes of the closely related *C. taiwanensis* LMG19424^T^ and “*C. neocaledonicus”* STM 6070 revealed that the pan- and variable genomes consisted of 5,205 and 438 genes, respectively, while 244 genes were unique to STM 6018 (Supplementary Figure S3). A previous progressiveMauve alignment of the draft genome of STM 6018 to the finished genome of LMG19424^T^ showed a high degree of synteny between the STM 6018 scaffolds and the LMG19424^T^ chromosome 1, chromosome 2 and symbiotic plasmid (Klonowska et al., 2020), suggesting that the STM 6018 replicons also consist of chromosome 1, chromosome 2 and a symbiotic plasmid.

The 244 genes unique to STM 6018 included two intact prophage regions, identified as a Mu-like phage and, surprisingly, a filamentous phage. Among the 5,205 genes that make up the *C. taiwanensis*/ “*C. neocaledonicus”* pangenome, the symbiotic, bacterial secretion system, and pilus system genes were highly conserved. Because these systems are important components of rhizobial interactions with legume hosts (Amadou et al., 2008; Deakin and Broughton, 2009; Zatakia et al., 2014), and of phage interactions with bacterial hosts, either for phage adsorption during infection (Bertozzi Silva et al., 2016; Hay and Lithgow, 2019), for secretion of filamentous phage virions from the host (Davis et al., 2000; Bille et al., 2005), or for secretion of phage-encoded toxins (Davis et al., 2000; Nakamura et al., 2021), we targeted them for further analysis. Below, we describe the STM 6018 symbiotic genotype and phenotype, secretion system and pilus system genes, and finally the two prophage genomes, concentrating on the filamentous phage, which has not previously been described in any rhizobial strain.

### Symbiotic genotype and phenotype of STM 6018

**Figure 1:**
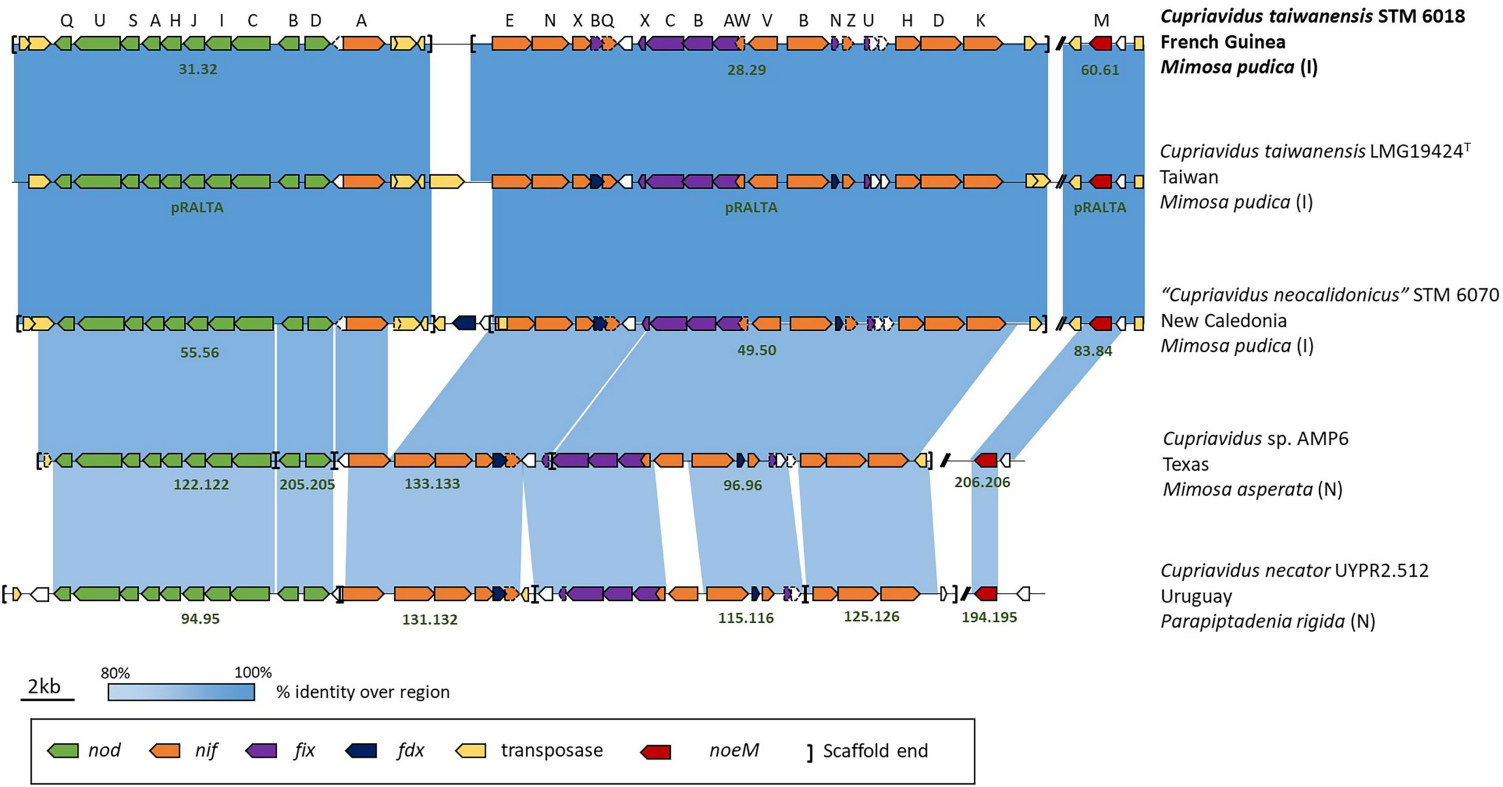
Schematic of the gene neighborhoods of symbiotic *Cupriavidus* species, showing nodulation (*nod*), nitrogen fixation (*nif, fdx* and *fix*) and transposase gene cluster alignments. Information on the microsymbiont’s host species, geographical location, and whether the host is introduced (I) or native (N) to the location is included. The scaffold number or replicon name is in dark green text and two bars (//) represent gaps in the sequence between the genes of interest.

The STM 6018, LMG19424^T^ and STM 6070 *nod*/*nif*/*noeM* gene clusters, along with characteristic mobile elements that are present in these clusters, were highly conserved in all three strains (Figure 1). Additional analysis of the nodulation (*nod* and *noe*) and nitrogen fixation (*nif*, *fix* and *fdx*) genes showed that these were well conserved in mimosoid-nodulating *Cupriavidus* strains (*C. taiwanensis* STM 6018 and LMG19424^T^, “*C. neocaledonicus”* STM 6070, *Cupriavidus* sp. AMP6, and *C. necator* UYPR2.512), with a high degree of both synteny and % identity (Figure 1). STM 6018, LMG19424^T^ and STM 6070 shared nearly 100% sequence identity (>99.97% bp identity over 100% coverage of the sequence of these genes), while AMP6 and UYPR2.512 shared lower % identities (>92% bp identity over >90% coverage and > 73% bp identity over >50% coverage, respectively) and lacked most of the transposase genes that are a feature of this gene neighborhood in the *C. taiwanensis* and “*C. neocaledonicus”* strains. This supports the hypothesis that symbiotic *Cupriavidus* populations have arisen *via* horizontal gene transfer (Parker, 2015) and suggests that the *M. pudica* isolates STM 6018, LMG19424^T^ and STM 6070 share well conserved symbiotic plasmids. All strains contained the nodulation genes *nodDBCIKHASUQ* and *noeM*, consistent with *C. taiwanensis* Nod factors being pentameric chito-oligomers with C18:1 or C16:0 fatty acyl chains, N-methylated and C-6 carbamoylated on the non-reducing end and sulfated on the reducing terminal residue, with additional production of atypical Nod factors with an open-chain oxidized terminal residue (Daubech et al., 2019). The recently described *noeM*, which is involved in the biosynthesis of these atypical Nod factors, is predominantly found in *M. pudica* microsymbionts and is important for symbiotic competitiveness of *C. taiwanensis* on *M. pudica* (Daubech et al., 2019). The putative NoeM proteins in STM 6018, STM 6070, AMP6 and UYPR2.512 were located and found to have high identity with the characterized NoeM of LMG19424^T^ (>70% identity in amino acid sequence, aligning >76% of the query protein). The rhizobial *Cupriavidus* nitrogen fixation genes were arranged in operons and included *nifA*, *nifENfdxBnifQ*, *nifX*, *nifVWfixABCX*, *nifBfdxNnifZfixU*, and *nifHDK* (Figure 1), as has been described for LMG19424^T^ (Amadou et al., 2008). Although these genes were all syntenic, the translated NifV of AMP6 and UYPR2.512 had low identity (77.89 and 48.13%, respectively) compared with the 100% identity shared by STM 6018, LMG19424^T^ and STM 6070 NifV (Figure 1). All five strains are capable of fixing nitrogen with *M. pudica* (Saad et al., 2012a; Klonowska et al., 2020), in accordance with the conservation of the *nod*, *nif*, *fix*, *fdx* and *noeM* gene regions.

*C. taiwanensis* STM 6018 forms nodules (Nod^+^) and effectively fixes N_2_ (Fix^+^) on a diverse range of *Mimosa* species. It proficiently fixes nitrogen with *Mimosa pudica* and *Mimosa pigra*, nodulates but does not fix nitrogen with *Mimosa caesalpiniaefolia* and *Mimosa acustipulata* and does not nodulate *Mimosa scabrella* (Table 2). Furthermore, STM 6018 has also been shown to out-compete a *gfp* marked derivative of *Paraburkholderia phymatum* STM815^T^ for the nodulation of *M. pudica* var. *unijuga* from Taiwan (80% nodule occupation), in contrast to the nodulation of *M. pudica* var. *tetrandra* (30% nodule occupation) or *hispida* (5% nodule occupation; Melkonian et al., 2014).

**Table 2:** Symbiotaxonomy of STM6018 using different species of *Mimosa*.

*Mimosa* species name	Seeds origin	Nod	Fix	Reference
*M. pudica var. unijuga*	Taiwan	+	+	Melkonian et al. (2014)
*M. pudica* var. *tetrandra*	French Guiana	+	+	Melkonian et al. (2014)
*M. pigra*	French Guiana	+	+	This study
*M. caesalpiniaefolia*	Brazil	+	−	This study
*M. acustipulata*	Brazil	+/−	−	This study
*M. scabrella*	Brazil	−	−	This study

Abbreviations: Nod (+), nodulates; Nod (−), no nodulation; Nod (+/−), only one nodule observed per plant (using triplicates); Fix (+), N_2_-fixing; Fix (−), no N_2_-fixing.

### STM 6018 secretion and pilus systems

Genomes of the *C. taiwanensis* strains STM 6018 and LMG19424^T^ contained loci encoding components of type I, II, III, IV and VI secretion systems and Type IV pilus systems (Supplementary Table S4), as well as loci for the Sec, Tat and SRP protein secretion systems.

### Type I secretion system (T1SS)

The T1SS is a heterotrimer composed of an outer membrane protein (TolC), a periplasmic membrane fusion protein (HlyD) and an inner membrane ATP-binding cassette transporter (HlyB; Kanonenberg et al., 2018). We identified five genes encoding the TolC outer membrane protein in the STM 6018 genome; four of these were associated with gene clusters that also contained *hlyB* and *hlyD* homologs (Supplementary Table S4). One cluster (A3AADRAFT_03604-A3AADRAFT_05738, syntenic with RALTA_B1439-RALTA_B1442) contained a gene encoding a putative exported metalloprotease with cadherin domains, while another cluster (A3AADRAFT_03425–03433, with no homolog in LMG19424^T^) contained sequences encoding a putative exported adhesin.

### Type II secretion system and type IV pilus systems

The T2SS is encoded by 12 core genes designated *gsp* (for General Secretory Pathway) and consists of a cytoplasmic hexameric ATPase (GspE); an inner membrane platform (GspC, GspF, GspL, GspM); a pseudopilus (GspG, GspH, GspI, GspJ, and GspK); a prepilin peptidase (GspO), required for proteolytic processing of the prepilin molecules, and an outer membrane pore (GspD), termed the secretin (Korotkov et al., 2012). In STM 6018, genes encoding the required T2SS components are clustered in a region (A3AADRAFT_04757–04770) that is syntenic with one on LMG19424^T^ chromosome 1 (RALTA_A2985-2,998). *gspO* was not present in this cluster, however, a gene encoding the prepilin peptidase homolog PilD was found in another region of the genome (A3AADRAFT_02062, RALTA_A2712) along with genes encoding the type IV pilus assembly proteins PilB and PilC (Supplementary Table S4). This appears to be similar to the arrangement in *Pseudomonas aeruginosa*, which requires PilD to process both type II and type IV pilin precursors (Nunn and Lory, 1992).

The TFP systems are evolutionarily related to the T2SS, having homologous components and similar architectures; specific types of TFP mediate adhesion, protein secretion, DNA uptake, and twitching motility, and are important for the formation of biofilms and in host colonization (Mattick, 2002; Denise et al., 2019). Bacterial pili also serve as the primary receptors of filamentous phage infection (Hay and Lithgow, 2019) – different phages capable of infecting the same organism adsorb specifically to different types of pilus during infection (Holland et al., 2006). STM 6018 and LMG19424^T^ contained six gene clusters encoding components of TFP systems (Supplementary Table S4). Two separate clusters (A3AADRAFT_01126–01139, RALTA_A0688-0702; A3AADRAFT_01206–01218, RALTA_B0189-0201) encoded components of a tight adherence (Tad) adhesive pilus system. The remaining clusters encoded separate components of a TFP system: the previously mentioned PilBCD, the alignment proteins PilMNOP and secretin PilQ (A3AADRAFT_04540–04544, RALTA_A2899-2,895), three clusters of the pilin proteins PilVWXYE and FimT, and the pilus retraction ATPases PilT and PilU (A3AADRAFT_02194–02195, RALTA_A2578-2,579). A monocistronic gene (A3AADRAFT_01884, RALTA_A0505) encoded the PilA major pilin. Pilus biogenesis *pil* loci are present on the symbiotic plasmid pRALTA of *C. taiwanensis* LMG 19424^T^ (Amadou et al., 2008) and on a syntenic region of STM 6018 (Supplementary Table S4). Genes encoding proteins involved in root attachment (*pilVWXYE*, *pilQPONM* and a monocistronic gene coding for a PilX-related protein) were shown to be up-regulated in LMG 19424^T^ cultures exposed to *M. pudica* root exudates (Klonowska et al., 2018), suggesting that they may play a role in host colonization.

### Type III secretion system

The T3SS is a complex nanomachine that injects effector proteins directly into the cytosol of host cells. T3SSs are important virulence determinants for bacterial pathogens and are also used by some rhizobial strains in symbiotic interactions with legume hosts (Marie et al., 2001). STM 6018 possesses a T3SS (A3AADRAFT_03420–03406) that is syntenic with that found on LMG19424^T^ chromosome 2 (RALTA_B1250–1,264; Amadou et al., 2008; Supplementary Table S4). We did not identify gene(s) in either STM 6018 or LMG19424^T^ encoding homologs of the SctA needle filament, or of the translocon that forms a pore in the host membrane through which effectors can enter, however, two hypothetical proteins within the T3SS gene cluster may be candidates for these roles (Supplementary Table S4). It has previously been shown that the T3SS of *C. taiwanensis* is not induced by *M. pudica* root exudates (Klonowska et al., 2018); moreover, regulation of *C. taiwanensis* T3SS genes was mediated by glutamate rather than legume flavonoids, and T3SS inactivation had no effect on *M. pudica* nodulation, but did allow nodulation and N_2_ fixation with *Leucaena leucocephala* (Saad et al., 2012b).

### Type IV secretion system

T4SSs are functionally diverse machineries for transporting DNA, proteins, or other macromolecules to bacterial or eukaryotic cell targets. There are two main subfamilies: (i) conjugation systems that mediate DNA transfer between bacterial cells, and (ii) translocators that deliver effector macromolecules into prokaryotic or eukaryotic cells (Costa et al., 2021). The *trb* and *tra* loci related to conjugative plasmid transfer are present on the symbiotic plasmid pRALTA of *C. taiwanensis* LMG 19424^T^ (Amadou et al., 2008) and on a syntenic region of STM 6018 (Supplementary Table S4).

### Type VI secretion system

The type VI secretion system (T6SS) is widely distributed among Gram-negative bacteria. It delivers effector toxins directly into a target cell and is usually deployed in competition against rival bacteria. The effector proteins are carried on a Vgr spike protein, which is fired into the target cell (Cianfanelli et al., 2016). We identified two separate T6SS systems in the genome of STM 6018, which were also present in LMG19424^T^. The first was on STM 6018 scaffold 0.1 in a neighborhood that was syntenic with that found on LMG19424^T^ chromosome 2 (Supplementary Table S4). In addition to the required T6SS machinery genes, this cluster contained a gene (locus tag A3AADRAFT_00194) encoding a novel type VI secretion protein, peptidoglycan L-alanyl-D-glutamate endopeptidase, which had 63% protein identity at the C-terminal end to the peptidoglycan-degrading enzyme TagX, required for type VI secretion in *Acinetobacter baumannii* (Weber et al., 2016). The second T6SS cluster, on STM 6018 scaffold 3.4 (LMG19424^T^ chromosome 1; Supplementary Table S4) was annotated as Sci-like proteins, from the T6SS cluster first identified in the *Salmonella enterica* subspecies I centisome 7 genomic island (Folkesson et al., 2002). The Vgr proteins all included RHS (rearrangement hot-spot) domains, often associated with type VI Vgr proteins as part of a protein secretion module with variable C-terminal toxic domains (Jackson et al., 2009). In addition to the *vgr* genes associated with these two clusters, a separate *vgr* gene (locus tag A3AADRAFT_01651, RALTA_A0273) was located upstream of genes encoding a putative effector protein – cognate immunity protein pair. STM 6018 also contained a *vgr* gene (A3AADRAFT_01451, scaffold 2.3) that was not found in LMG19424^T^.

### Characterization of the STM 6018 Mu-like prophage genome

**Figure 2:**
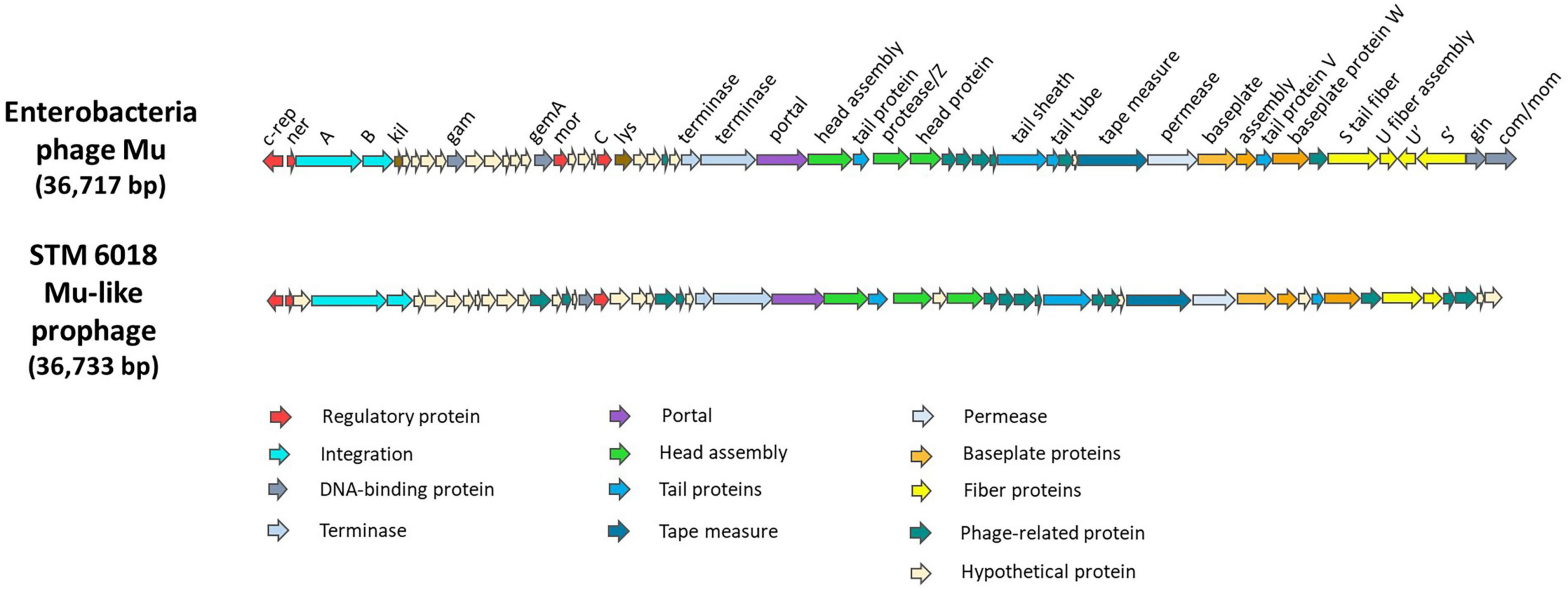
Genes and genome organization of Enterobacteria phage Mu (Morgan et al., 2002) and the Mu-like phage found in STM 6018.

The intact Mu-like prophage was identified on scaffold 19.20 of STM 6018, inserted between a gene encoding an acyl-CoA dehydrogenase (locus tag A3AADRAFT_05251) and a gene encoding salicylate hydroxylase (locus tag A3AADRAFT_05309). The gene neighborhoods on each side of the Mu-like prophage are syntenic with those found on LMG19424^T^ chromosome 2. Bacteriophage Mu is a member of the Myoviridae family of tailed bacteriophages and has a number of distinctive properties, including the ability to integrate into nearly random chromosomal locations (Morgan et al., 2002). The STM 6018 Mu-like prophage had an estimated size of 36,733 bp with a similar genome architecture to Mu and contained most of the core genes (Morgan et al., 2002) but had low core protein identity (25–40%) and lacked homologs to several core genes, including those encoding the G-segment invertase Gin, the adenine modification enzyme Mom, and an invertible segment encoding tail fibers (Figure 2). NCBI BLASTN analysis of the complete STM 6018 Mu-like prophage region identified similar prophages in the genomes of several other *C. taiwanensis* strains. LMG 19424^T^ lacks this Mu-like prophage but instead has previously been characterized as containing a prophage on Chromosome 1 that is similar to the Phi CTX phage of *P. aeruginosa* (Amadou et al., 2008).

### Characterization of the STM 6018 filamentous prophage genome

Although a recent study has highlighted the diversity of filamentous phages and their presence in a broad range of bacterial and archaeal hosts (Roux et al., 2019), and filamentous phages have been reported to infect *Sinorhizobium* (*Ensifer*) *meliloti* (Cubo et al., 2020), to the best of our knowledge this is the first characterization of a filamentous prophage from the genome of a rhizobial strain. Filamentous phages currently belong to the *Inoviridae* family. Well-studied examples include the *Escherichia coli* Ff phages (M13, fd and F1; Marvin and Hoffmann-Berling, 1963), the *Vibrio cholerae* CTXϕ phage (Waldor and Mekalanos, 1996), and the Pf phages of *P. aeruginosa* (Knezevic et al., 2015). Filamentous phages replicate by a rolling circle mechanism (Baas, 1985) and possess several unique morphological and genetic features. Within the host cell, they can exist as either episomes or as integrated prophages, and can replicate without causing cell death, as viral particles are secreted across the cell envelope rather than released *via* lysis (Marvin, 1998). Whereas most bacteriophages have double-stranded DNA genomes packaged within a protein capsid, the virions of filamentous phages are long filaments, with circular, single-stranded DNA genomes of ~5–15 kb surrounded by several thousand major coat protein subunits arranged in a helical array (Mai-Prochnow et al., 2015; Hay and Lithgow, 2019).

**Figure 3:**
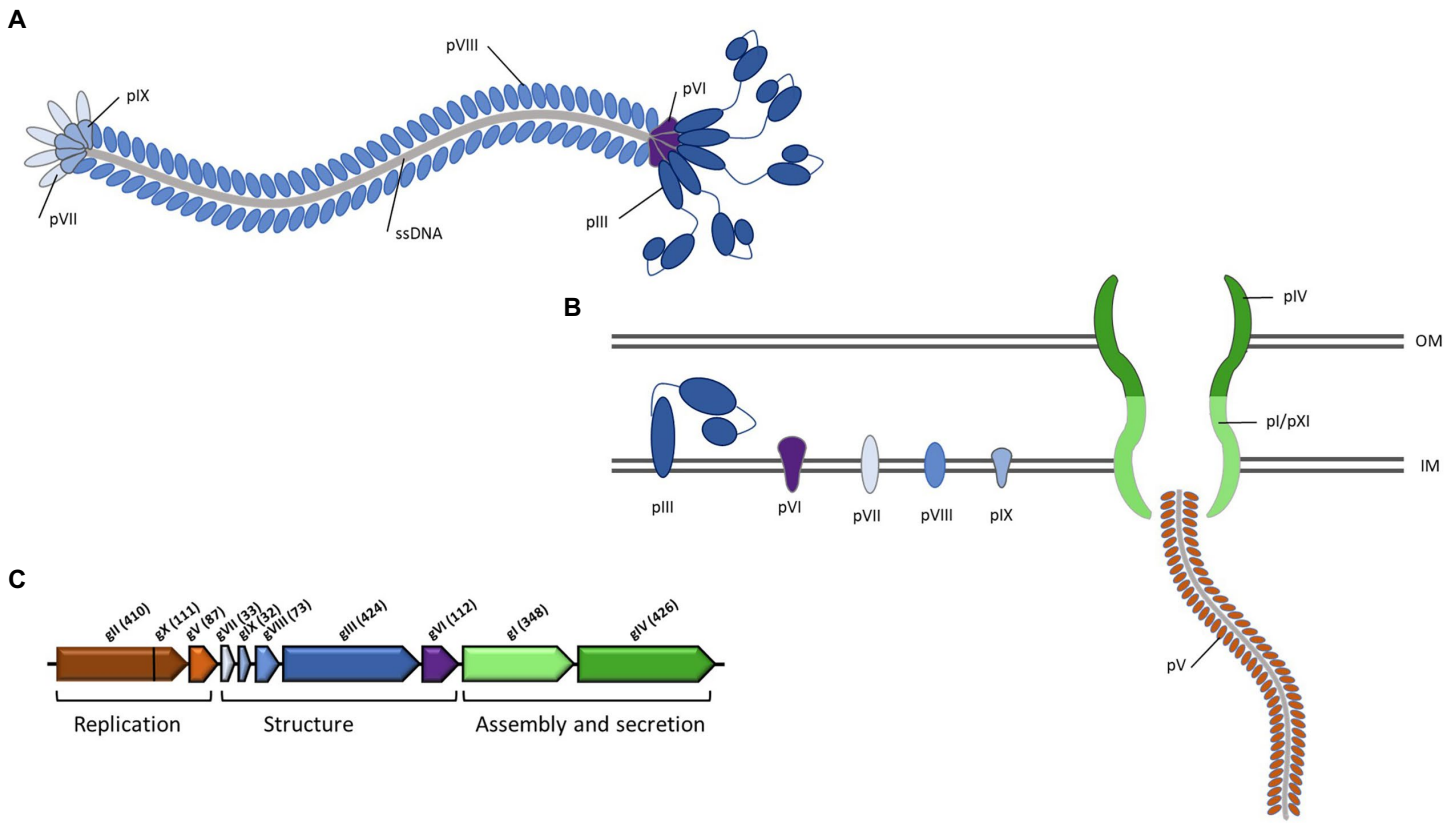
General schema of genes and genome organization of filamentous phages. The genes and the proteins they encode are color-coded according to function, where brown indicates replication; blue indicates structural; green indicates assembly and secretion. **(A)** the mature virion; **(B)** the virion within the bacterial host cell; associated phage structural, assembly and secretion proteins are embedded within the bacterial membrane; **(C)** genome organization of the model Ff filamentous phage, with the standard gene notations of gI to gX. Figure adapted from Mai-Prochnow et al. (2015).

The genomes of filamentous phages are modularly organized, mosaic structures. The encoded proteins have low identity across different phages, however, the order of the core genes and their sizes and membrane topology tends to be conserved, allowing putative identification of the genes, based on those of the well-studied Ff phages of *E. coli* (Mai-Prochnow et al., 2015). Figure 3 shows a general diagram of a filamentous phage, the phage genome, genes, and encoded proteins. The core genes include modules for (1) replication (R), (2) structural components (S), and (3) assembly and secretion [AS; reviewed in Mai-Prochnow et al. (2015) and Hay and Lithgow (2019)]. R genes encode the pII protein that is required for DNA replication, the pV protein that binds to phage ssDNA in the cytoplasm and the pX protein that binds to dsDNA and prevents hydrolysis. pX is translated from an internal start site within the pII gene. S proteins include the major coat protein pVIII and the minor coat proteins pIII, pVI, pVII and pIX. pIII additionally plays an essential role in host infection, as it specifically mediates adsorption of the phage, firstly to the host cell pilus primary receptor and then to the TolA component of the inner membrane TolQRA complex, which forms part of the Tol-Pal complex involved in cell division and membrane integrity. S proteins are all integrated into the host inner membrane. The AS module includes pI, pIV and pXI. These proteins form a trans-envelope complex that is essential for assembly and secretion of the phage, where pI/pXI form the inner membrane component and pIV is an outer membrane secretin that belongs to the same family as type II and type III outer membrane channels and the type IV pilus assembly system. pXI is translated from an internal start site within the pI gene. pXI and pIV are not always conserved in filamentous phages, and in cases where the gene encoding pIV is absent, a host-encoded secretin is used instead. pI contains ATP-binding Walker A and Walker B motifs and is presumed to be an ATPase that powers the assembly and transport of the phage out of the bacterial cell (Loh et al., 2017). Regulatory genes may also be present in the phage genome, as well as accessory genes. A notable example is the CTXϕ phage, where accessory genes encode the CtxAB toxin, a primary virulence factor that converts *V. cholerae* strains carrying the phage to deadly pathogens (Waldor and Mekalanos, 1996). Only the pI protein gene is universally conserved across all members of the *Inoviridae* (Roux et al., 2019).

**Figure 4:**
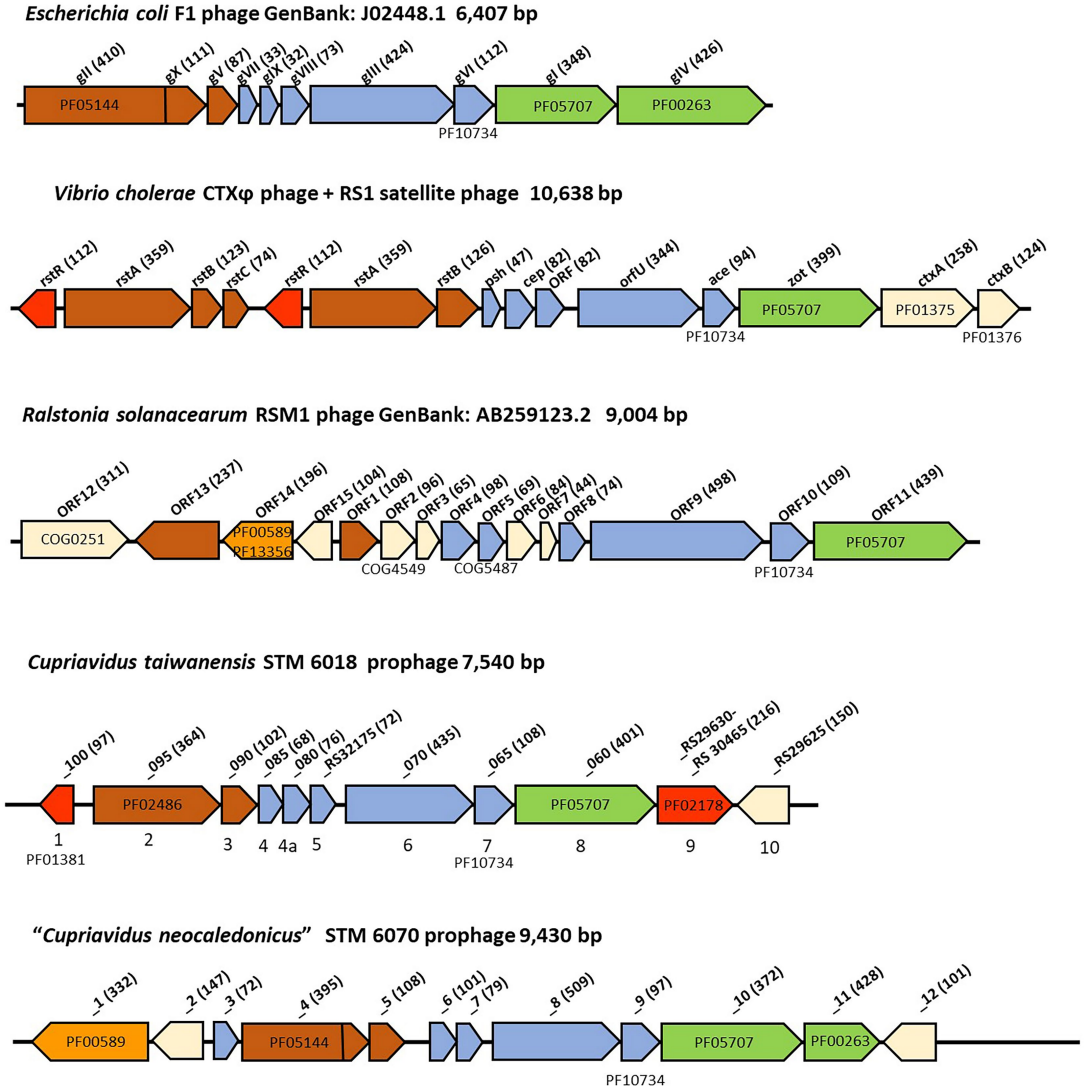
Comparison of filamentous phage genomes. The genes are colored according to function, where red indicates regulatory genes, brown indicates replication genes, orange indicates genes encoding integrases, blue indicates structural genes, and green indicates assembly and secretion genes. White indicates genes that are unique for each phage. The amino acid sequence length of the encoded proteins is in brackets. Where relevant, COG and pfam numbers of the encoded proteins are shown.

The filamentous prophage identified in the genome of STM 6018 was located on scaffold 0.1, in a region that was syntenic with that of *C. taiwanensis* LMG 19424^T^ chromosome II. The prophage genome has an estimated size of 7,540 bp in length, has a GC content of 61.1% and includes 11 predicted protein-encoding genes along with flanking regions, based on the annotations in IMG and NCBI (Supplementary Table S4). Based on these annotations and the order, size and membrane topology of the core genes, we identified the putative replication, structural, assembly and secretion, regulatory and accessory genes in the STM 6018 prophage, and compared its genome with those of other well-characterized filamentous phages (Mai-Prochnow et al., 2015; Figure 4; Supplementary Table S4).

Genes encoding putative pII, pV, pVII, pIX, pVIII, pIII, pVI and pI proteins were all present, however, as is typical with filamentous phages, they had low sequence identity (< 30%) with the core proteins of characterized filamentous phages (Mai-Prochnow et al., 2015; Supplementary Table S4). There are some notable differences between the STM 6018 filamentous phage and the *E. coli* F1 phage (Figure 4). The STM 6018 filamentous phage lacks a gene encoding the pIV secretin and therefore presumably relies on a host secretin to transport virions out of the cell, as has been found for *V. cholerae* and *Neisseria meningitidis* filamentous phages (Davis et al., 2000; Bille et al., 2005). The putative pII protein was identified as DNA relaxase NicK (Rep_trans endonuclease pfam02486) rather than the phage replication protein CRI (pfam05144) present in Ff phages; moreover, the STM 6018 phage pII lacked a domain that was functionally equivalent to the pX protein. Rolling circle replication initiator proteins are known to be diverse (Wawrzyniak et al., 2017), as demonstrated by the various pII proteins found in characterized filamentous phages (Figure 4).

**Figure 5:**
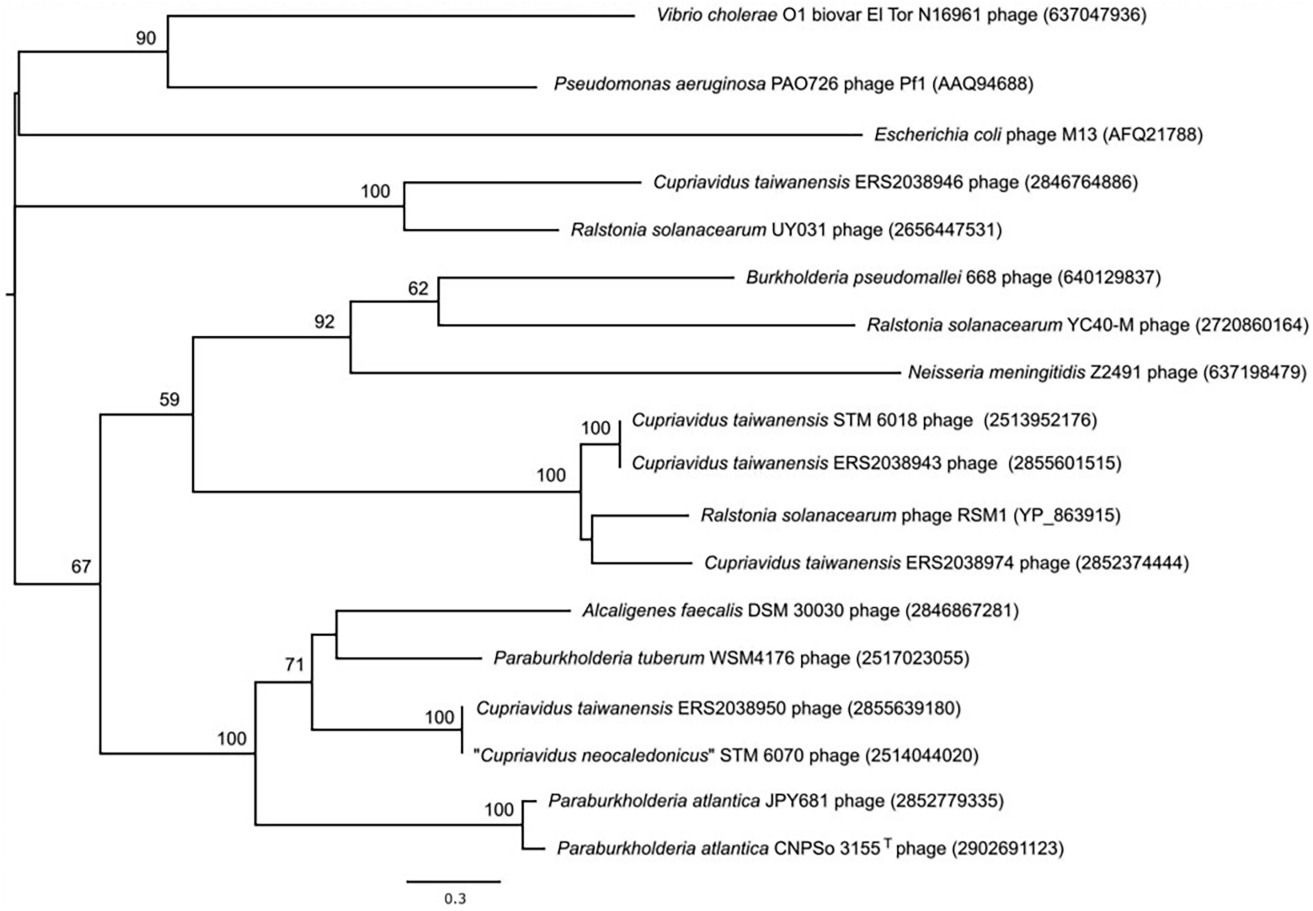
Phylogenetic tree based on the amino acid sequences of pI (zonula occludens toxin) proteins of filamentous phages from the genomes of *Cupriavidus taiwanensis* STM 6018 and diverse bacterial animal and plant pathogens, symbionts, and environmental strains. NCBI and IMG accession numbers are given in parenthesis. A total of 547 amino acid positions were used to reconstruct the tree. The evolutionary history was inferred by using the Maximum Likelihood method and a discrete Gamma distribution was used to model evolutionary rate differences among sites. Human pathogens, or potentially pathogenic species in the tree include *Burkholderia pseudomallei*, *Escherichia coli*, *Neisseria meningitidis*, *Pseudomonas aeruginosa* and *Vibrio cholerae*; *Ralstonia solanacearum* is a plant pathogen; the *Cupriavidus* and *Paraburkholderia* spp. are rhizobial symbionts; and *Alcaligenes faecalis* is an environmental species.

The pI proteins in some pathogenic *V. cholerae* and *Campylobacter concisus* strains are additional virulence determinants, due to cleavage and secretion of a toxic C-terminal domain that binds to tight junctions (zonula occludens) between intestinal epithelial cells and increases permeability (Fasano et al., 1991; Mahendran et al., 2016), causing pI proteins to be generally annotated as zonula occludens toxins (Zot). Because rhizobial strains are generally classified as Risk Group 1 (organisms that do not cause disease in healthy adult humans) and because they are often used as inoculants for legume crops and pastures, we examined the sequence of the STM6018 phage pI protein for the presence of this toxic domain. However, the C-terminus of *V. cholerae* Zot that contains the biologically active fragment (FCIGRL; Di Pierro et al., 2001) is missing in the pI of the STM 6018 filamentous prophage. and a phylogenetic tree of pI proteins from phages of characterized bacterial animal and plant pathogens, symbionts, and environmental strains places the STM 6018 pI protein well away from the *V. cholerae* Zot (Figure 5). Similarly, the CTXϕ protein Ace (accessory cholera enterotoxin) that is functionally equivalent to the pVI protein (Waldor and Mekalanos, 1996) is an additional *V. cholerae* virulence determinant that activates calcium-dependent chloride-bicarbonate secretion (Trucksis et al., 1993, 2000) but this role in virulence has not been reported for any other filamentous phage pVI protein. The STM 6018 prophage also contains accessory genes encoding a transcriptional regulator (A3AADRAFT_00018), a putative regulator that contains a DNA-binding AT-hook motif (A3AADRAFT_00010), and a hypothetical protein (A3AADRAFT_0009; Figure 4).

The AT-hook (pfam02178) is a small DNA-binding motif that contains a core GRP or Q/RGR sequence. It binds to the minor groove of AT-rich DNA regions and is found in many eukaryotic nuclear proteins (Aravind and Landsman, 1998) and in bacterial nucleoid-associated proteins such as H-NS (histone-like nucleoid structuring protein) from some Gram-negative bacteria and Lsr2 from mycobacteria (Gordon et al., 2011). However, the STM 6018 prophage AT-hook motif protein is unrelated to H-NS or Lsr2 proteins and moreover is structurally different, as the canonical RGR motif is found in the N-terminal region of the protein instead of the N-terminal oligomerization domain and AT-hook C-terminal DNA binding domain of H-NS- and Lsr2-like proteins (Gordon et al., 2011; Pfeifer et al., 2016). BLASTp analysis indicated that this protein is conserved in several filamentous prophages, as we identified genes encoding similar proteins containing an AT-hook motif located downstream of the gene encoding the pI protein in the genomes of various *Cupriavidus* and *Ralstonia* strains.

Although the STM 6018 filamentous prophage does not contain homologs of H-NS-like proteins, *C. taiwanensis* does contain several genes annotated in IMG as encoding H-NS or H-NS-like proteins (pfam00816; Supplementary Table S4). H-NS and Lsr2 specifically target and downregulate gene expression from AT-rich regions of DNA (Gordon et al., 2011; Singh et al., 2016). This includes genes acquired by horizontal gene transfer, which are relatively AT-rich compared with the rest of the bacterial genome and need to be tightly regulated to prevent fitness costs from their unregulated expression. H-NS and Lsr2 therefore play significant roles as xenogenic silencers of this foreign DNA, facilitating integration of horizontally acquired genes into the host regulatory network and contributing to bacterial evolution (Navarre, 2016). Xenogenic silencing also appears to be required for the survival of bacterial cells that contain prophages. In *P. aeruginosa*, loss of the xenogenic silencing proteins MvaT and MvaU, which are functionally equivalent to H-NS, results in an increase in gene expression and subsequent increase in the production of the filamentous phage Pf4, leading to cell death or inhibition of cell growth (Castang and Dove, 2012). Recent papers have also identified genes encoding H-NS- and Lsr2-like proteins in phage genomes, with either predicted roles in repressing host defense mechanisms against phage infection (Skennerton et al., 2011) or a demonstrated role in silencing gene expression of the prophage and consequent maintenance of the lysogenic state within the host genome (Pfeifer et al., 2016). We postulate that the AT-hook motif-containing protein encoded by A3AADRAFT_00010 may play a similar role in regulating expression of either host or prophage genes.

Because filamentous prophages have not previously been described in rhizobia, we evaluated their occurrence in sequenced genomes of genera that are known to contain rhizobial strains, using the conserved pI (Zot) protein (COG4128, pfam05707) as a marker for the presence of *Inoviridae* prophages (Roux et al., 2019) and then further establishing whether the gene encoding the Zot domain was within an intact prophage. We identified genes that encoded a Zot domain in the genomes of some alphaproteobacterial strains, however, these proteins were small (79–142 amino acids) and the genes were not associated with a prophage. In contrast, we identified intact filamentous prophages in rhizobial and non-rhizobial strains of betaproteobacterial *Cupriavidus, Paraburkholderia* and *Trinickia*. A total of 13 of these genomes were rhizobial strains (Supplementary Table S5). The phylogenetic tree of these pI proteins (Figure 5) demonstrates that highly diverse prophages can infect a given bacterial species, as shown here by the *C. taiwanensis* prophages. Rhizobial strains that contained intact filamentous prophages included the well-characterized “*C. neocaledonicus*” STM 6070 (Klonowska et al., 2020) and *Paraburkholderia tuberum* WSM4176 (Howieson et al., 2013; Reeve et al., 2015). We therefore selected the prophages of these strains for further analysis, with an emphasis on the prophages within the two *Cupriavidus* genomes.

### Comparison of the filamentous prophages in STM 6070 and WSM4176 with the STM 6018 prophage

The STM 6070 filamentous prophage was located on scaffold 19.20 in a region that was syntenic with LMG 19424^T^ Chromosome 1 and STM 6018 scaffold 9.10. There was very low sequence identity between the prophage found in STM 6018 and that of STM 6070; moreover, the STM 6070 prophage genome contained more genes, had a different gene order for some of the core phage proteins and had a different pII protein to that of the STM 6018 filamentous prophage (Figure 4; Supplementary Table S4). The genome included genes encoding a XerD-like site-specific tyrosine recombinase of the phage integrase family, a pIV secretin and two hypothetical proteins (Figure 4; Supplementary Table S4). The pII phage replication protein had the same pfam (pfam05144) as that of the pII CRI protein of Ff phages and similarly included a pX domain within the pII gene.

The *P. tuberum* WSM4176 prophage was located on scaffold 3.7 and similarly had low sequence identity with the STM 6018 and STM 6070 prophages. In addition to the core genes, the genome included genes encoding a Lambda repressor-like DNA-binding domain, a pIV secretin and three hypothetical proteins (Supplementary Table S4). The pII phage replication protein was a DNA relaxase NicK (pfam02486).

### The STM 6018 and STM 6070 phages integrate into different sites within the *Cupriavidus* genome

**Figure 6:**
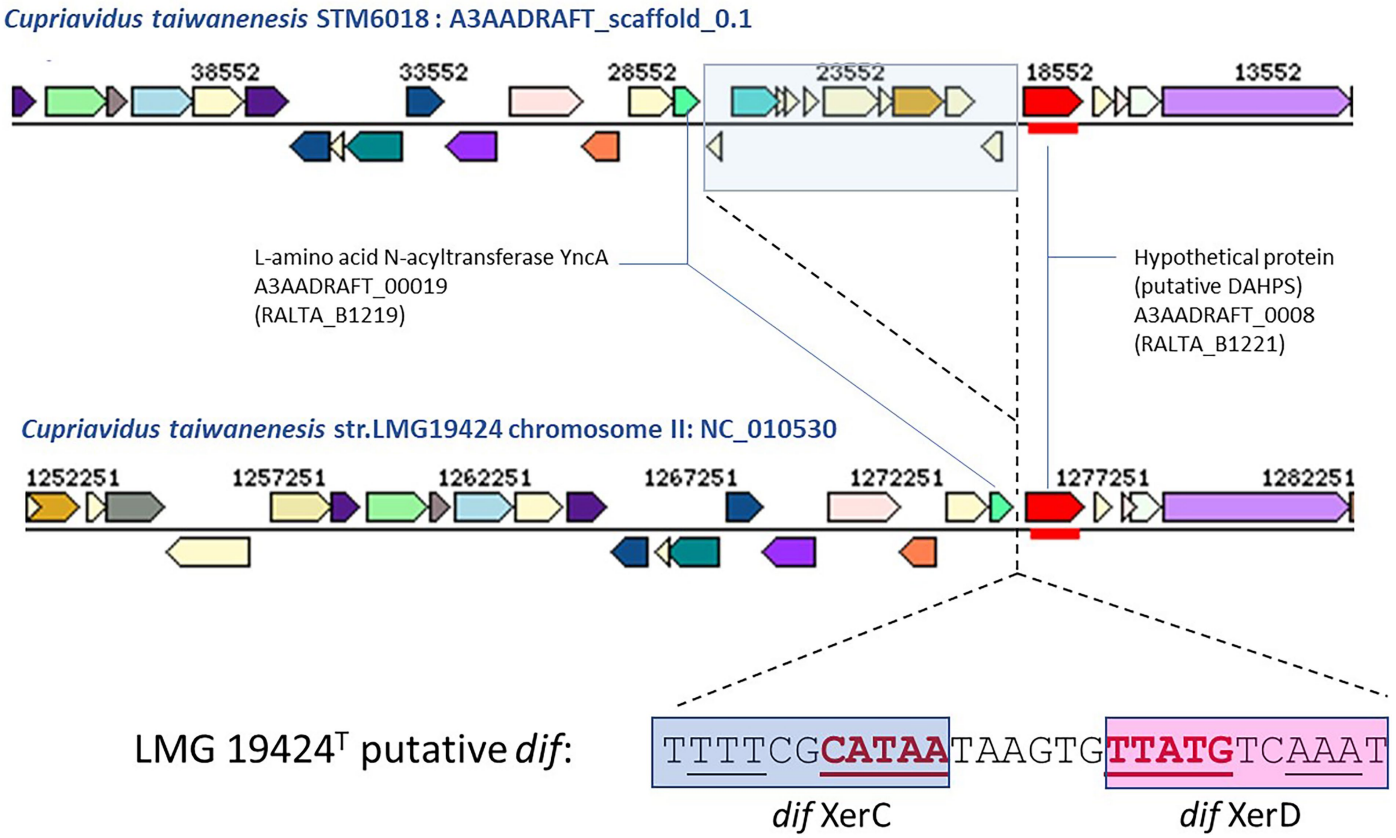
Comparison of the gene neighborhoods of *Cupriavidus taiwanensis* strains STM 6018 and LMG 19424^T^ showing the site of integration of the filamentous phage found in STM 6018 and the DNA sequence of the putative *dif* site and Xer binding sites. Xer binding sites were identified using the highly conserved XerD binding sites of closely related *Ralstonia* spp. reported by Carnoy and Roten (2009).

To further characterize the STM 6018 and STM 6070 prophages, we examined where they integrated in the *Cupriavidus* genome. Integration of filamentous phages into host chromosomes occurs by site specific recombination and employs diverse strategies, according to the type of phage. Characterized phages may use *dif*, tRNA or inverted repeat target sequences and either host- or phage-encoded recombination or transposase systems (Hay and Lithgow, 2019). In *C. taiwanensis*, the STM 6018 prophage located at position 19,342–26,881 on scaffold 0.1 is integrated into a putative *dif* (deletion induced filamentation) site between genes encoding L-amino acid N-acyltransferase YncA (A3AADRAFT_00019; = RALTA_B1219 in LMG19424^T^) and a putative 3-deoxy-d-arabino-heptulosonate 7-phosphate synthase (DAHPS; A3AADRAFT_0008; = RALTA_B1221 in LMG19424^T^; Figure 6). This region lacks synteny with STM 6070, as the genes encoding YncA and putative DAHPS homologs are found on separate scaffolds in the STM 6070 genome. The *dif* site is normally the site where resolution of concatenated chromosomes occurs *via* XerC and XerD site-specific recombination, but is also used by various filamentous phages for integration/excision (Carnoy and Roten, 2009). In STM 6018, the left-hand and right-hand flanking regions of the filamentous prophage contain DNA sequences that are homologous to the *C. taiwanensis dif* sequence (Figure 6). The STM 6018 filamentous phage lacks genes encoding Xer tyrosine recombinases and presumably relies on the host XerCD to integrate into the chromosome, which is the mechanism employed by *V. cholerae* CTXϕ (McLeod and Waldor, 2004). The STM 6018 genome contains several genes encoding XerC and XerD homologs (Supplementary Table S4). In contrast, the STM 6070 filamentous phage integrates into a tRNA-Met site within STM 6070, presumably *via* its phage-encoded XerD-like site-specific tyrosine recombinase. Similarly, characterized *Pseudomonas* phages integrate into tRNA-Gly, −Met and -Sec sites (Fiedoruk et al., 2020), while the *Ralstonia* phage RSM1 reversibly integrates into a tRNA-Ser site (Hay and Lithgow, 2019).

### Effects of phage infection on bacterial hosts and symbiotic implications

Filamentous phages can have marked effects on the virulence of their pathogenic bacterial hosts. A well-known example is *V. cholerae*, where strains that acquire the CTXϕ phage become toxigenic due to phage-encoded cholera toxin (Waldor and Mekalanos, 1996). Filamentous phages have also been shown to affect biofilm formation, exopolysaccharide (EPS) biosynthesis, motility, bacterial cell aggregation and expression of virulence genes, which can either enhance or reduce bacterial colonization, infectivity, or pathogenicity of a eukaryote host [reviewed in Mai-Prochnow et al. (2015)]. For example, *Ralstonia solanacearum* cells infected with the ϕRSS1 phage had enhanced virulence on tobacco and tomato plants, whereas cells infected with φRSM-type phages showed loss of virulence (Askora and Yamada, 2015). Similarly, other types of prophage can have significant effects on bacterial interactions, and may provide ecological and evolutionary benefits (Harrison and Brockhurst, 2017). Indeed, it has been suggested that the relationship between filamentous phages and their bacterial hosts, rather than being parasitic, can be one of facultative mutualism, with fitness benefits to the host in certain environments (Shapiro et al., 2016). Little is currently known of the effects of phage infection on rhizobial hosts. However, as biofilms, EPS, motility and expression of symbiotic genes are important for rhizobial lifestyles (Rinaudi and Giordano, 2010), we suggest that if phage infection causes changes in these attributes it will affect rhizobial ecology and relationships with legume hosts. More widely, it has been proposed that the tripartite relationship between phages, bacteria, and plants be explored further due to the putative effects of filamentous phages in improving the fitness of bacterial inocula to promote ecorestoration (Sharma et al., 2019). As the legume-rhizobia symbiosis is of particular importance in the restoration of degraded ecosystems (Chen et al., 2008; Teng et al., 2015; Jach et al., 2022), further research should be performed to explore the role of filamentous prophages in rhizobial colonization and competition for nodulation of the host plant, ecological fitness, and bacterial population structure. STM 6018 and other rhizobial strains that contain filamentous prophages will be useful resources in this enterprise.

## Materials and methods

### Growth conditions, genomic DNA isolation and nodulation, and phenotypic assay

*C. taiwanensis* STM 6018 was isolated from a soil sample from French Guiana, South America, as previously described, using the trap host *M. pudica* (Mishra et al., 2012). Bacterial isolates were cultured on ½LA (Howieson et al., 1988), TY (Beringer, 1974) or YMA (Howieson and Dilworth, 2016) at 28°C. For long-term maintenance, bacterial strains were grown in YM broth and preserved in 20% glycerol at −80°C.

For bacterial genomic DNA isolation *C*. *taiwanensis* STM 6018 was streaked onto TY solid medium and grown at 28°C for 3 days to obtain well grown separated colonies, and then a single colony was selected and used to inoculate 5 ml TY broth medium. The culture was grown for 48 h on a gyratory shaker (200 rpm) at 28°C. Subsequently, 1 ml was used to inoculate 60 ml TY broth medium that was incubated on a gyratory shaker (200 rpm) at 28°C until an OD600nm of 0.6 was reached. DNA was isolated from 60 ml of cells using a CTAB bacterial genomic DNA isolation method (Howieson and Dilworth, 2016). Final concentration of the DNA was set to 0.5 mg ml^−1^.

For nodulation assay of *Mimosa* spp. plants seedlings were prepared as previously described by Mishra et al. (2012) and were inoculated 7 days after germination with 1 ml of bacterial cell suspensions from cultures in exponential growth phase, as described in Melkonian et al. (2014).

### Genome sequencing and assembly

The genome of *C. taiwanensis* STM 6018 was sequenced at the JGI using the Illumina technology (Bennett, 2004). An Illumina standard shotgun library was constructed and sequenced using the Illumina HiSeq 2000 platform which generated 14,977,300 reads totaling 2,245 Mb. Genome sequencing project information is shown in Supplementary Table S6.

All raw Illumina sequence data was passed through DUK, a filtering program developed at JGI, which removes known Illumina sequencing and library preparation artifacts (Mingkun, L., Copeland, A. and Han, J.).^1^ The following steps were then performed for assembly: (1) filtered Illumina reads were assembled using Velvet (Zerbino, 2010; version 1.1.04), (2) 1–3 Kb simulated paired end reads were created from Velvet contigs using wgsim,^2^ (3) Illumina reads were assembled with simulated read pairs using Allpaths–LG (Gnerre et al., 2011; version r39750). Parameters for assembly steps were: (1) Velvet (−-v --s 51 --e 71 --i 2 --t 1 --f “-shortPaired -fastq $FASTQ” --o “-ins_length 250 -min_contig_lgth 500” 10) (2) wgsim (−e 0–1 76–2 76 –r 0 –R 0 –X 0 3) Allpaths–LG (PrepareAllpathsInputs:PHRED64 = 1 PLOIDY = 1 FRAGCOVERAGE = 125 JUMPCOVERAGE = 25 LONGJUMPCOV = 50, RunAllpath-sLG: THREADS = 8 RUN = stdshredpairs TARGETS = standard VAPIWARNONLY = True OVERWRITE = True).

### Genome annotation

Genes were identified using Prodigal (Hyatt et al., 2010) as part of the JGI annotation pipeline (Huntemann et al., 2015). The predicted CDSs were translated and used to search the National Center for Biotechnology Information (NCBI) nonredundant database,^3^ TIGRFams database,^4^ UniProt,^5^ Pfam (now hosted by InterPro),^6^ PRIAM (Claudel-Renard et al., 2003), KEGG,^7^ COG,^8^ and InterPro^9^ databases. The tRNAScanSE tool (Lowe and Eddy, 1997) was used to find tRNA genes, whereas ribosomal RNA genes were found by searches against models of the ribosomal RNA genes built from SILVA (Pruesse et al., 2007). Other non–coding RNAs such as the RNA components of the protein secretion complex and the RNase P were identified by searching the genome for the corresponding Rfam profiles using INFERNAL.^10^ Additional gene prediction analysis and manual functional annotation was performed within the Integrated Microbial Genomes (IMG-ER) platform^11^ (Markowitz et al., 2009) and the genome was released through the Integrated Microbial Genomes System (Chen et al., 2021).

### Genome analyses

The species assignment for STM 6018 was assessed by calculating the Average Nucleotide Identity (ANI) values of this genome to taxonomically proximal *Cupriavidus* and *Ralstonia* genomes. ANIb or ANIm values were computed with jSpecies (Richter and Rosselló-Móra, 2009) using either alignments produced by BLASTN or MUMmer, respectively. ANIg values were also computed as pairwise bidirectional best nSimScan hits of genes having 70% or more identity and at least 70% coverage of the shorter gene (Varghese et al., 2015). Species affiliation cut-off used was set at >95% ANI over 69% of the conserved DNA for ANIb or ANIm values (Goris et al., 2007) or 96.5% for ANIg values (Varghese et al., 2015).

The comparison of gene orthologs of STM 6018 with those of the symbiotic *Cupriavidus* strains LMG 19424^T^ and STM 6070 was performed using the “Gene Phyloprofile” tool in the Microscope MaGe platform (Vallenet et al., 2019). The orthologous counterparts in the genomes were detected by applying parameters of a minimum of 30% for protein sequence identity over a minimum of 80% of the protein length (>30% protein MinLrap 0.8). Additional searches for homologous proteins were performed using the BLAST program in IMG with parameters of >30% protein sequence identity over a minimum of 80% of the protein length.

To identify the presence of prophages within other rhizobial genomes, we used the pI protein (COG4128, pfam05707) as a marker for the presence of *Inoviridae* prophages and searched for this marker within the genomes of both Alpha- and Betaproteobacterial rhizobial genera within the IMG database, using the Function Search tool in IMG. We then examined the gene neighborhoods of genes encoding a pfam05707 domain to determine whether the gene was within an intact filamentous prophage.

### Phylogenetic analyses

We assessed the phylogenetic position of STM 6018 using a 1,301 bp internal region of the 16S rRNA gene. *Ralstonia solanacearum* LMG2299^T^ was used as an outgroup. Sequences were aligned using MUSCLE as implemented in MEGA, version X (Kumar et al., 2018). Phylogenetic analyses were performed in MEGA X (Kumar et al., 2018) using the Neighbor-Joining method (Saitou and Nei, 1987). The evolutionary distances were computed using the Maximum Composite Likelihood method (Tamura et al., 2004) and are in the units of the number of base substitutions per site. Bootstrap analysis (Felsenstein, 1985) with 1,000 replicates was performed to assess the support of the clusters.

We analyzed the phylogeny of the pI protein from the STM 6018 filamentous prophage using the amino acid sequences of characterized phage pI proteins and translated amino acid sequences of the pI gene identified in filamentous prophages present in Betaproteobacterial rhizobial genomes. Sequences were aligned using MUSCLE as implemented in MEGA, version X (Kumar et al., 2018). The evolutionary history was inferred using the Maximum Likelihood method based on the Le_Gascuel_2008 model (Le and Gascuel, 2008). The tree with the highest log likelihood (−13502.17) is shown. Initial tree(s) for the heuristic search were obtained automatically by applying Neighbor-Join and BioNJ algorithms to a matrix of pairwise distances estimated using a JTT model, and then selecting the topology with superior log likelihood value. A discrete Gamma distribution was used to model evolutionary rate differences among sites [5 categories (+G, parameter = 5.0459)]. A total of 547 amino acid positions were used in the final dataset. Evolutionary analyses were conducted in MEGA X (Kumar et al., 2018).

## Data availability statement

The datasets presented in this study can be found in online repositories. The names of the repository/repositories and accession number(s) can be found in the article/Supplementary material.

## Author contributions

AK isolated and performed the initial study of the STM 6018 strain. WR coordinated the project and supplied the DNA to the JGI for sequencing. All authors were involved in sequencing, annotation and analysis of the genome and/or editing the final paper. JA, LM, AK, JZ, and WR drafted the manuscript. All authors contributed to the article and approved the submitted version.

## Funding

The work (proposal: 10.46936/10.25585/60007492) conducted by the US Department of Energy Joint Genome Institute (https://ror.org/04xm1d337), a DOE Office of Science User Facility, is supported by the Office of Science of the US Department of Energy operated under Contract No. DE-AC02-05CH11231.

## Conflict of interest

The authors declare that the research was conducted in the absence of any commercial or financial relationships that could be construed as a potential conflict of interest.

## Publisher’s note

All claims expressed in this article are solely those of the authors and do not necessarily represent those of their affiliated organizations, or those of the publisher, the editors and the reviewers. Any product that may be evaluated in this article, or claim that may be made by its manufacturer, is not guaranteed or endorsed by the publisher.

## Footnotes

^1^ https://www.osti.gov/biblio/1016000

^2^ https://github.com/lh3/wgsim

^3^ https://www.ncbi.nlm.nih.gov/

^4^ https://www.ncbi.nlm.nih.gov/genome/annotation_prok/tigrfams/

^5^ https://www.uniprot.org/

^6^ https://www.ebi.ac.uk/interpro/search/text/398365647/#table

^7^ https://www.genome.jp/kegg/

^8^ https://www.ncbi.nlm.nih.gov/research/cog-project/

^9^ https://www.ebi.ac.uk/interpro/

^10^ http://infernal.janelia.org

^11^ http://img.jgi.doe.gov/er

## Abbreviations

½LA: 1/2 strength Lupin Agar
ANI: Average Nucleotide Identity
DoE: Department of Energy
EPS: Exopolysaccharide
GEBA-RNB: Genomic Encyclopedia for Bacteria and Archaea-Root Nodule Bacteria
GOLD: Genomes OnLine Database
MIGS: Minimum Information about the Genome Sequence
TY: Tryptone-Yeast extract agar
YMAm: Yeast-Mannitol Agar (modified)
