# Supplementary material for: Discovery of a novel filamentous prophage in the genome of the Mimosa pudica microsymbiont Cupriavidus taiwanensis STM 6018
Source: Front Microbiol. 2023 Feb 28;14:1082107. doi: 10.3389/fmicb.2023.1082107 (PMC10011098; doi:10.3389/fmicb.2023.1082107)
Supplement: Supplementary file 1 [file Table_1.docx]

Table S1. Classification and general features of *Cupriavidus taiwanensis* STM6018 in accordance with the MIGS recommendations [1]

| **MIGS ID** | **Property** | **Term** | **Evidence code**^a^ |
| --- | --- | --- | --- |
|  | Current classification | Domain Bacteria | TAS [2] |
|  |  | Phylum *Proteobacteria* | TAS [3] |
|  |  | Class *Betaproteobacteria* | TAS [4] |
|  |  | Order *Burkholderiales* | TAS [5] |
|  |  | Family *Burkholderiaceae* | TAS [6] |
|  |  | Genus *Cupriavidus* | TAS [7] |
|  |  | Species *taiwanensis* | TAS [8] and this study |
|  |  |  |  |
|  | Gram stain | Negative | IDA |
|  | Cell shape | Rod | IDA |
|  | Motility | Motile | IDA |
|  | Sporulation | Non-sporulating | NAS |
|  | Temperature range | Mesophile | NAS |
|  | Optimum temperature | 28°C | NAS |
|  | Salinity | Non-halophile | NAS |
| MIGS-22 | Oxygen requirement | Aerobic | IDA |
|  | Carbon source | Varied | NAS |
|  | Energy source | Chemoorganotroph | NAS |
| MIGS-6 | Habitat | Soil, root nodule, on host | TAS [11] |
| MIGS-15 | Biotic relationship | Free living, symbiotic | TAS [11] |
| MIGS-14 | Pathogenicity | Non-pathogenic | NAS |
|  | Biosafety level | 1 | TAS [9] |
|  | Isolation | Root nodule of *Mimosa pudica* | TAS [9] |
| MIGS-4 | Geographic location | Roadside of the Remire town in French Guinea | TAS [9] |
| MIGS-5 | Soil collection date | November 2006 | IDA |
| MIGS-4.1 MIGS-4.2 | Longitude  Latitude | -52.312778  4.880833 | TAS [11] |
| MIGS-4.3 | Depth | 0-10 cm | NAS |
| MIGS-4.4 | Altitude | 7 m | TAS [9] |

^a^ Evidence codes – IDA: Inferred from Direct Assay; TAS: Traceable Author Statement (i.e., a direct report exists in the literature); NAS: Non-traceable Author Statement (i.e., not directly observed for the living, isolated sample, but based on a generally accepted property for the species, or anecdotal evidence). These evidence codes are from http://www.geneontology.org/GO.evidence.shtml of the Gene Ontology project [10].

**References**

1. Field, D., Garrity, G., Gray, T., Morrison, N., Selengut, J., Sterk, P., Tatusova, T., Thomson, N., Allen, M., Angiuoli, S.V., Ashburner, M., Axelrod, N., Baldauf, S., Ballard, S., Boore, J.L., Cochrane, G., Cole, J., Dawyndt, P., De Vos, P., De Pamphilis, C., Edwards, R., Faruque, N., Feldman, R., Gilbert, J., Gilna, P., Glöckner, F.O., Goldstein, P., Guralnick, R., Haft, D., Hancock, D., Hermjakob, H., Hertz-Fowler, C., Hugenholtz, P., Joint, I., Kagan, L., Kane, M., Kennedy, J., Kowalchuk, G., Kottmann, R., Kolker, E., Karvitz, S., Kyripides, N., Leebens-Mack, J., Lewis, S., Li, K., Liste, A., Lord, P., Maltsev, N., Markowitz, V., Martiny, J., Methe, B., Moxon, R., Nelson, K., Parkhill, J., Proctor, L., Sansone, S., Spiers, A., Stevens, R., Swift, P., Taylor, C., Tateno, Y., Tett, A., Turner, S., Ussery, D., Vaughan, B., Ward, N., Whetzel, T., Wilson, G., and Wipat, A. (2008). Towards a richer description of our complete collection of genomes and metagenomes "Minimum Information about a Genome Sequence " (MIGS) specification. *Nature Biotechnology* 26**,** 541-547.

2. Woese, C.R., Kandler, O., and Wheelis, M.L. (1990). Towards a natural system of organisms: proposal for the domains Archaea, Bacteria, and Eucarya. *Proceedings of the National Academy of Sciences of the United States of America* 87, 4576-4579.

3. Garrity, G.M., Bell, J.A., and Lilburn, T. (2005a). "Phylum XIV. Proteobacteria phyl. nov.," in *Bergey's Manual of Systematic Bacteriology,* eds. G.M. Garrity, D.J. Brenner, N.R. Kreig & J.T. Staley. Second ed: New York: Springer - Verlag), 1.

4. Garrity, G.M., Bell, J.A., and Lilburn, T.E. (2005b). "Class II. Betaproteobacteria," in *Bergey's Manual of Systematic Bacteriology,* eds. G.M. Garrity, D.J. Brenner, N.R. Krieg & J.T. Staley. Second ed (New York: Springer - Verlag).

5. Garrity, G.M., Bell, J.A., and Lilburn, T.E. (2005d). "Order 1. *Burkholderiales*," in *Bergey's Manual of Systematic Bacteriology,* eds. G.M. Garrity, D.J. Brenner, N.R. Krieg & J.T. Staley. Second ed (New York: Springer - Verlag).

6. Garrity, G.M., Bell, J.A., and Lilburn, T.E. (2005c). "Family I. Burkholderiaceae," in *Bergey's Manual of Systematic Bacteriology,* eds. G.M. Garrity, D.J. Brenner, N.R. Krieg & J.T. Staley. Second ed (New York: Springer - Verlag).

7. Vandamme, P., and Coenye, T. (2004). Taxonomy of the genus *Cupriavidus*: a tale of lost and found. *International Journal of Systematic and Evolutionary Microbiology* 54**,** 2285-2289.

8. Chen, W.M., Laevens, S., Lee, T.M., Coenye, T., De Vos, P., Mergeay, M., and Vandamme, P. (2001). *Ralstonia taiwanensis* sp. nov., isolated from root nodules of *Mimosa* species and sputum of a cystic fibrosis patient. *International Journal of Systematic and Evolutionary Microbiology* 51**,** 1729-1735.

9. Mishra, R.P., Tisseyre, P., Melkonian, R., Chaintreuil, C., Miché, L., Klonowska, A., Gonzalez, S., Bena, G., Laguerre, G., and Moulin, L. (2012). Genetic diversity of *Mimosa pudica* rhizobial symbionts in soils of French Guiana: investigating the origin and diversity of *Burkholderia phymatum* and other beta-rhizobia. *FEMS Microbiology Ecology* 79, 487-503.

10. Ashburner, M., Ball, C.A., Blake, J.A., Botstein, D., Butler, H., Cherry, J.M., Davis, A.P., Dolinski, K., Dwight, S.S., Eppig, J.T., Harris, M.A., Hill, D.P., Issel-Tarver, L., Kasarskis, A., Lewis, S., Matese, J.C., Richardson, J.E., Ringwald, M., Rubin, G.M., and Sherlock, G. (2000). Gene Ontology: Tool for the unification of biology. The Gene Ontology Consortium. *Nature Genetics* 25**,** 25-29.
