# Supplementary material for: Discovery of a novel filamentous prophage in the genome of the Mimosa pudica microsymbiont Cupriavidus taiwanensis STM 6018
Source: Front Microbiol. 2023 Feb 28;14:1082107. doi: 10.3389/fmicb.2023.1082107 (PMC10011098; doi:10.3389/fmicb.2023.1082107)
Supplement: Supplementary file 2 [file Table_2.DOCX]

**Table S2.** Genome statistics for *Cupriavidus taiwanensis* STM6018

| **Attribute** | **Value** | **% of Total** | | |
| --- | --- | --- | --- | --- |
| Genome size (bp) | 6,553,639 | | 100.00 |  |
| DNA coding region (bp) | 5,753,902 | | 87.80 |  |
| DNA G+C content (bp) | 4,384,459 | | 66.90 |  |
| Number of scaffolds | 80 | |  |  |
| Number of contigs | 80 | |  |  |
| Total gene | 5925 | | 100.00 |  |
| RNA genes^1^ | 61 | | 1.03 |  |
| Protein-coding genes | 5864 | | 98.97 |  |
| Protein coding genes with function prediction | 4781 | | 80.69 |  |
| Protein coding genes assigned to COGs | 4324 | | 72.98 |  |
| Protein coding genes assigned Pfam domains | 5044 | | 85.13 |  |
| Protein coding genes with signal peptides | 663 | | 11.19 |  |
| Protein coding genes with transmembrane helices | 1411 | | 23.81 |  |
| Chromosomal cassettes | 602 | |  |  |
| Biosynthetic clusters | 8 | |  |  |
| CRISPR repeats | 1 | |  |  |
| Fused Protein coding genes | 65 | | 1.10 |  |
| Protein coding genes coding signal peptides | 663 | | 11.19 |  |
| Protein coding genes coding transmembrane proteins | 1411 | | 23.81 |  |

^1^four copies of 5S rRNA, two copies of 16S and one copy of 23S.
