# Supplementary material for: Discovery of a novel filamentous prophage in the genome of the Mimosa pudica microsymbiont Cupriavidus taiwanensis STM 6018
Source: Front Microbiol. 2023 Feb 28;14:1082107. doi: 10.3389/fmicb.2023.1082107 (PMC10011098; doi:10.3389/fmicb.2023.1082107)
Supplement: Supplementary file 3 [file Table_3.DOCX]

**Table S3.** Number of protein coding genes of *Cupriavidus taiwanensis* STM6018 associated with the general COG functional categories.

| **Code** | **COG Category with extra row at the beginning** | **Gene Count** | **% of total (4,324)** |
| --- | --- | --- | --- |
|  | CELLULAR PROCESSES AND SIGNALING |  |  |
| D | Cell cycle control, cell division, chromosome partitioning | 34 | 0.69 |
| M | Cell wall/membrane/envelope biogenesis | 282 | 5.72 |
| N | Cell motility | 116 | 2.35 |
| O | Posttranslational modification, protein turnover, chaperones | 175 | 3.55 |
| T | Signal transduction mechanisms | 237 | 4.81 |
| U | Intracellular trafficking, secretion, and vesicular transport | 108 | 2.19 |
| V | Defense mechanisms | 108 | 2.19 |
| W | Extracellular structures | 54 | 1.10 |
| Z | Cytoskeleton | 1 | 0.02 |
|  | INFORMATION STORAGE AND PROCESSING |  |  |
| A | RNA processing and modification | 1 | 0.02 |
| B | Chromatin structure and dynamics | 3 | 0.06 |
| J | Translation, ribosomal, structure and biogenesis | 222 | 4.50 |
| K | Transcription | 453 | 9.19 |
| L | Replication, recombination and repair | 133 | 2.70 |
|  | METABOLISM |  |  |
| C | Energy production and conversion | 476 | 9.65 |
| E | Amino acid transport and metabolism | 425 | 8.62 |
| F | Nucleotide transport and metabolism | 96 | 1.95 |
| G | Carbohydrate transport and metabolism | 226 | 4.58 |
| H | Coenzyme transport and metabolism | 228 | 4.62 |
| I | Lipid transport and metabolism | 343 | 6.96 |
| P | Inorganic ion transport and metabolism | 274 | 5.56 |
| Q | Secondary metabolite biosynthesis, transport and catabolism | 192 | 3.89 |
|  | POORLY CHARACTERIZED |  |  |
| R | General function prediction only | 471 | 9.55 |
| S | Function unknown | 221 | 4.48 |
| X | Mobilome, prophages, transposons | 52 | 1.05 |
|  | Not in COGs | 1601 | 27.02 |
