# Supplementary material for: Discovery of a novel filamentous prophage in the genome of the Mimosa pudica microsymbiont Cupriavidus taiwanensis STM 6018
Source: Front Microbiol. 2023 Feb 28;14:1082107. doi: 10.3389/fmicb.2023.1082107 (PMC10011098; doi:10.3389/fmicb.2023.1082107)
Supplement: Supplementary file 5 [file Table_5.docx]

Table S5. Rhizobial strains in the Integrated Microbial Genomes (IMG) database with identified filamentous prophages within their genomes. The IMG locus tag of the zonula occludens toxin gene (*zot*) used as a marker to identify filamentous prophages within the genomes is listed.

| **Genus species** | **Strain** | **Host Legume** | **Geographical Location** | **IMG Locus Tag of *zot*** | **Reference** |
| --- | --- | --- | --- | --- | --- |
| *“Cupriavidus neocaledonicus”* | STM 6070 | *Mimosa pudica* | New Caledonia | A3AGDRAFT_04113 | Klonowska et al., 2020 |
| *Cupriavidus taiwanensis* | ERS2038943 | ND* | ND | Ga0399869_01_2004055_2005260 | ND |
| *C. taiwanensis* | ERS2038946 | ND | ND | Ga0398238_12_5831_6961 | ND |
| *C. taiwanensis* | ERS2038950 | ND | ND | Ga0398548_01_2422686_2423804 | ND |
| *C. taiwanensis* | ERS2038966 | ND | ND | Ga0398997_01_1798562_1799758 | ND |
| *C. taiwanensis* | ERS2038969 | ND | ND | Ga0398997_01_1798562_1799758 | ND |
| *C. taiwanensis* | ERS2038971 | ND | ND | Ga0399298_02_1524088_1525290 | ND |
| *C. taiwanensis* | ERS2038974 | ND | ND | Ga0398117_01_2541138_2542328 | ND |
| *C. taiwanensis* | STM 6018 | *Mimosa pudica* | French Guiana | A3AADRAFT_00011 | This study |
| *Paraburkholderia atlantica* | CNPSo 3155^T^ | *Mimosa pudica* | Brazil | Ga0444200_225_80712_81824 | Paulitsch et al., 2020 |
| *P. atlantica* | JPY681 | *Mimosa somnians* | Mexico | Ga0400115_05_198818_199933 | Bontemps et al., 2015 |
| *Paraburkholderia tuberum* | WSM4176 | *Lebeckia ambigua* | South Africa | Ga0537054_01_2232_3356 | Howieson et al., 2013 |
| *P. tuberum* | WSM4179 | *Lebeckia ambigua* | South Africa | B014DRAFT_04850 | Howieson et al., 2013 |

*Not determined; genome has not been published.

**References**

Bontemps, C., Rogel, M.A., Wiechmann, A., Mussabekova, A., Moody, S., Simon, M.F., Moulin, L., Elliott, G.N., Lacercat-Didier, L., Dasilva, C., Grether, R., Camargo-Ricalde, S.L., Chen, W., Sprent, J.I., Martínez-Romero, E., Young, J.P.W., and James, E.K. (2015). Endemic *Mimosa* species from Mexico prefer alphaproteobacterial rhizobial symbionts. *New Phytologist* 209**,** 319-333. doi: 10.1111/nph.13573

Howieson, J.G., De Meyer, S.E., Vivas-Marfisi, A., Ratnayake, S., Ardley, J.K., and Yates, R.J. (2013). Novel *Burkholderia* bacteria isolated from *Lebeckia ambigua* – a perennial suffrutescent legume of the fynbos. *Soil Biology and Biochemistry* 60**,** 55-64. doi: 10.1016/j.soilbio.2013.01.009

Klonowska, A., Moulin, L., Ardley, J.K., Braun, F., Gollagher, M.M., Zandberg, J.D., Marinova, D.V., Huntemann, M., Reddy, T., and Varghese, N.J. (2020). Novel heavy metal resistance gene clusters are present in the genome of *Cupriavidus neocaledonicus* STM 6070, a new species of *Mimosa pudica* microsymbiont isolated from heavy-metal-rich mining site soil. *BMC Genomics* 21**,** 1-18. doi: 10.1111/j.1574-6941.2012.01393.x

Paulitsch, F., Dall’agnol, R.F., Delamuta, J.R.M., Ribeiro, R.A., Da Silva Batista, J.S., and Hungria, M. (2020). *Paraburkholderia atlantica* sp. nov. and *Paraburkholderia franconis* sp. nov., two new nitrogen-fixing nodulating species isolated from Atlantic forest soils in Brazil. *Archives of Microbiology* 202**,** 1369-1380. doi: 10.1016/j.syapm.2020.126152
