# Supplementary material for: Discovery of a novel filamentous prophage in the genome of the Mimosa pudica microsymbiont Cupriavidus taiwanensis STM 6018
Source: Front Microbiol. 2023 Feb 28;14:1082107. doi: 10.3389/fmicb.2023.1082107 (PMC10011098; doi:10.3389/fmicb.2023.1082107)
Supplement: Supplementary file 6 [file Table_6.DOCX]

**Table S5.** Genome sequencing project information for *Cupriavidus taiwanensis* STM6018.

| **Property** | **Term** | **MIGS ID** |
| --- | --- | --- |
| Finishing quality | Improved high-quality draft | MIGS-31 |
| Libraries used | Illumina Standard Paired End | MIGS-28 |
| Sequencing platforms | Illumina HiSeq 2000 | MIGS-29 |
| Sequencing coverage | 449x | MIGS-31.2 |
| Assemblers | ALLPATHS v. R37654 | MIGS-30 |
| Gene calling methods | Prodigal 2.5 | MIGS-32 |
| GOLD Project ID | Gp0009785 |  |
| NCBI BioProject ID | 165307 |  |
| Database: IMG | 2513237150 |  |
| Project relevance | Symbiotic N_2_ fixation, agriculture |  |
