# Supplementary figures and images for: Discovery of a novel filamentous prophage in the genome of the Mimosa pudica microsymbiont Cupriavidus taiwanensis STM 6018
Source: Front Microbiol. 2023 Feb 28;14:1082107. doi: 10.3389/fmicb.2023.1082107 (PMC10011098; doi:10.3389/fmicb.2023.1082107)

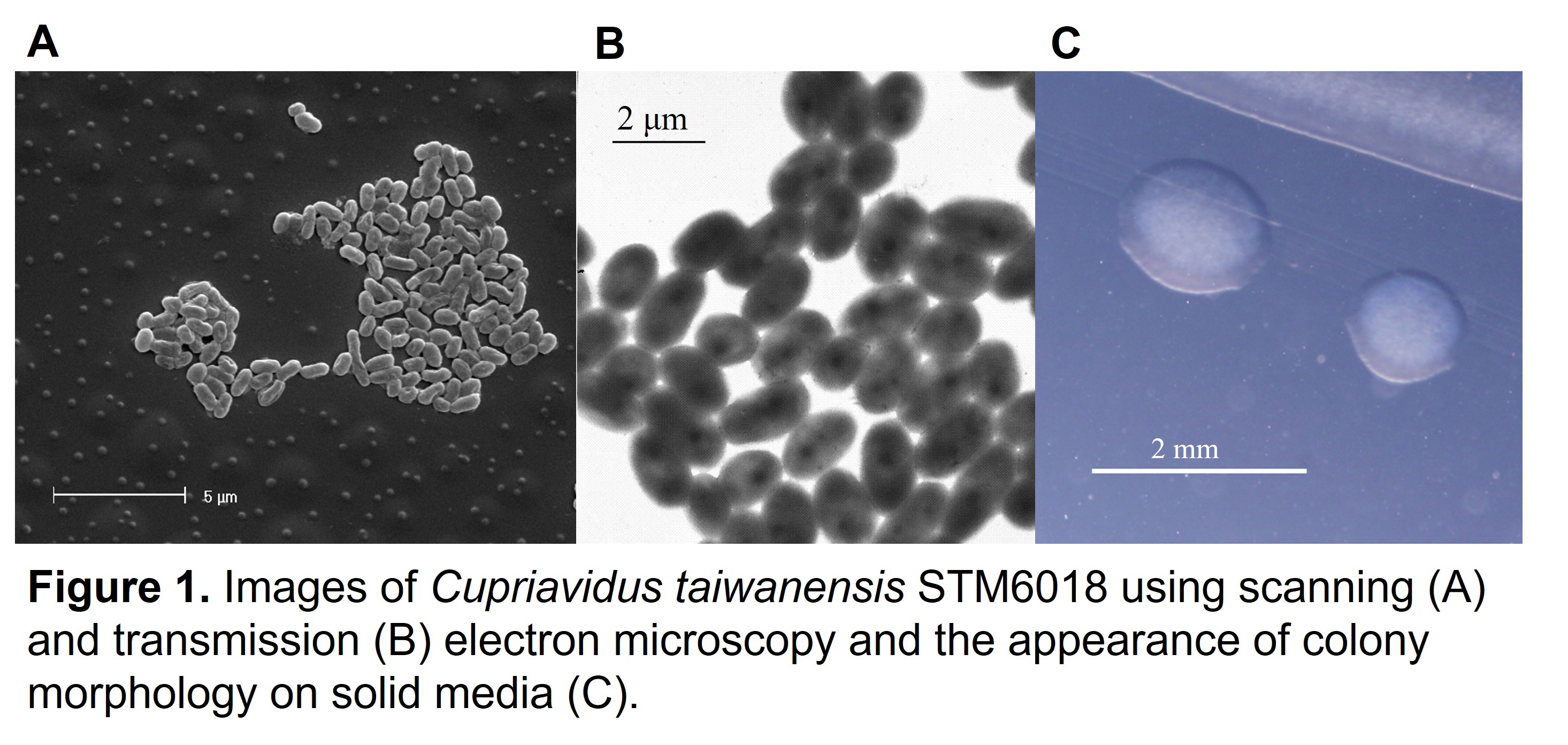

Supplement: Supplementary file 7 [file Image_1.JPEG]

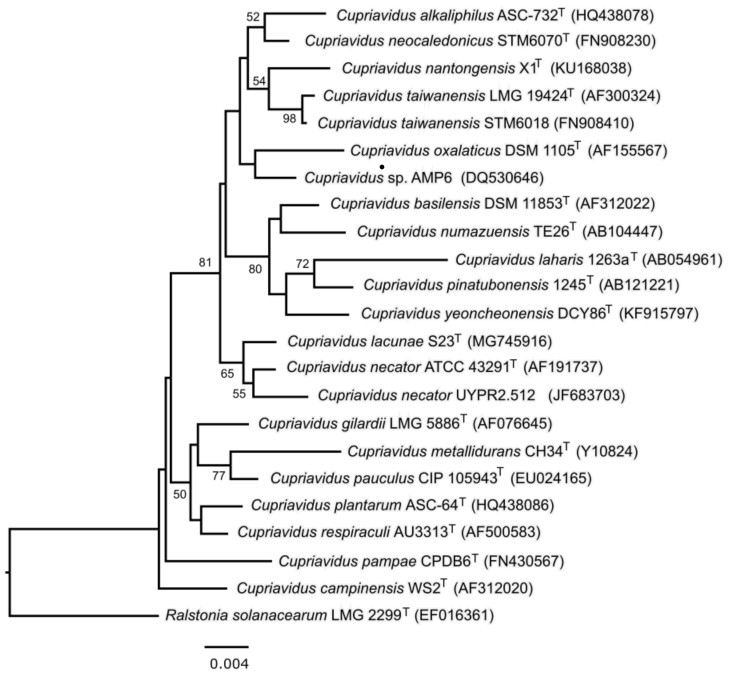

Supplement: Supplementary file 8 [file Image_2.JPEG]

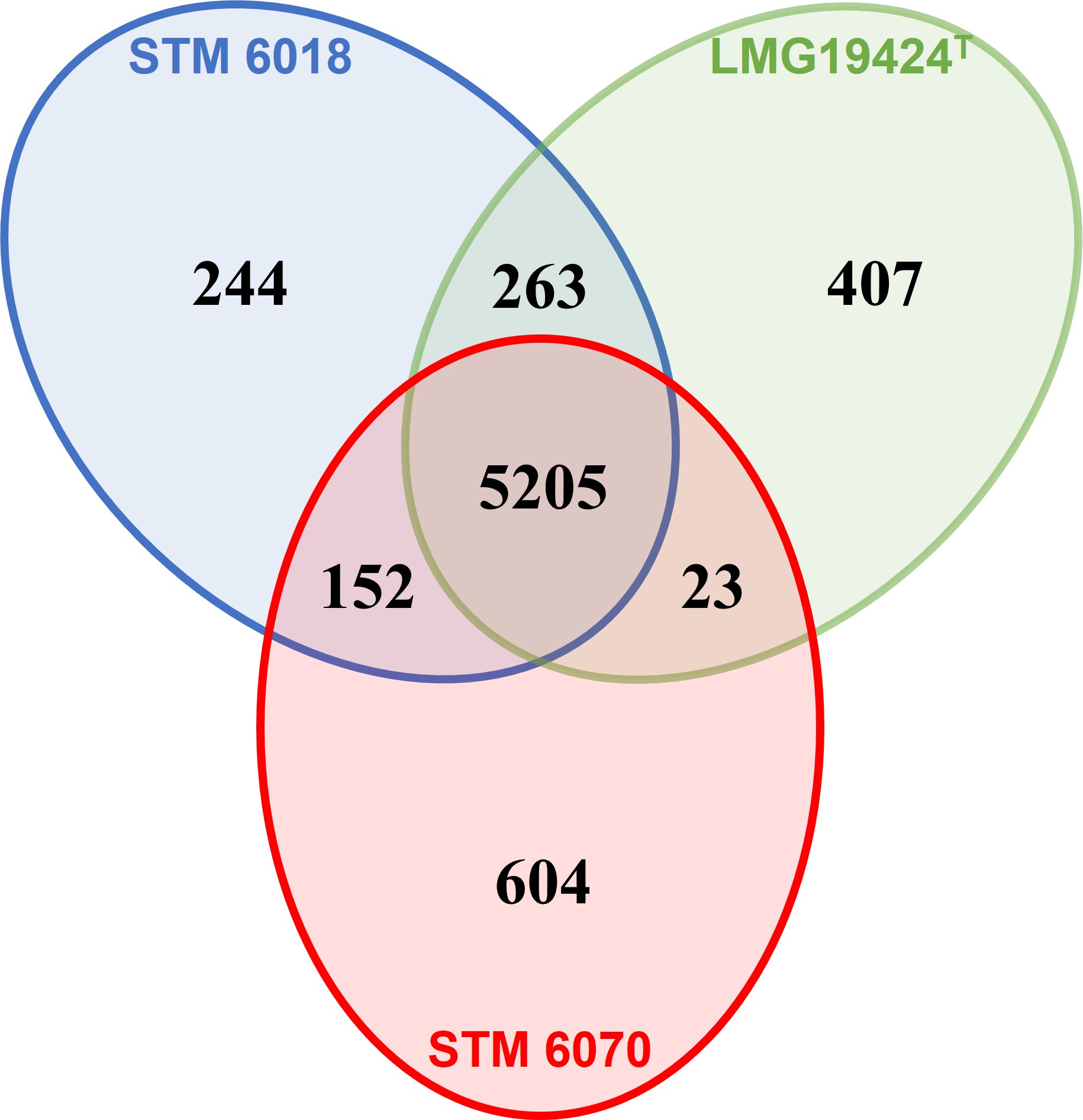

Supplement: Supplementary file 9 [file Image_3.JPEG]
